# The transgender patient in primary care: practical advice for a 10-minute consultation

**DOI:** 10.3399/bjgpopen17X101001

**Published:** 2017-10-04

**Authors:** Charlotte Cliffe, Miriam Hillyard, Albert Joseph, Azeem Majeed

**Affiliations:** 1 Honorary Clinical Research Fellow, Department of Primary Care and Public Health, Imperial College London, London, UK; 2 Foundation Doctor, North West Thames Foundation School, Imperial College Healthcare NHS Trust, London, UK; 3 Honorary Clinical Research Fellow, Department of Primary Care and Public Health, Imperial College London, London, UK; 4 Professor of Primary Care, Department of Primary Care and Public Health, Imperial College London, London, UK

**Keywords:** transgender, gender identity, gender variance, bridging prescriptions

With referrals to gender identity clinics rising rapidly, GPs are more likely to meet patients who are transgender (whose gender identity, or internal sense of gender, does not match their gender assigned at birth) or diagnosed with gender dysphoria (the severe psychological distress that is experienced by an individual as a result of the conflict between their gender identity and gender assigned at birth).^1^ Teaching on transgender medicine is lacking in both undergraduate and postgraduate curricula, leading to a perceived lack of expertise in this area. Furthermore, General Medical Council (GMC) guidelines on the GP’s role in prescribing are vague, resulting in some controversy. As wait times for appointments at specialist clinics are often at least 18 months, primary care physicians will increasingly be involved in the initiation of the transition process: this is the process by which an individual changes their phenotypic appearance of gender to match their gender identity through medications and/or surgery.

## Example case

A 36-year-old patient, who was male at birth, has been living as a woman for the last 9 months. Her birth name still appears on GP records. She would like legal recognition as a woman and a referral to specialist gender services.

### What needs to be ascertained?

#### Preferred pronoun and name

The patient's preferred name and pronoun should be updated on the electronic patient record system; this will help prevent any potential mistakes from being made by calling the patient by the wrong name or gender.^2^

#### Level of support

Gather an understanding of the individual's relationship with family, friends, or a wider social network (including transgender communities) to determine their level of support. Social isolation, negative reactions of family and friends, or limited ‘social transition’ can be risk factors for developing mental illness or gender dysphoria, both of which may require extra support from mental health teams.^2^

#### Mental health symptoms

Elicit information regarding any distress, anxiety, self-harm, or suicidal thoughts. Around 34% of transgender individuals have attempted suicide at least once; screening in this initial consultation is important as it helps to determine whether a referral to the community mental health team is required.^3^

#### Self-medication

Patients may obtain unregulated and unverified hormonal medications; these are usually procured online.^4^ These could:

- contain contaminants; 
- have doses different from those stated; or
- be inactive.

GPs should determine whether there are any absolute contraindications to hormonal preparations; such as, past history of thrombosis, breast cancer, or current pregnancy. There are also relative contraindications that should be considered and for which specialist advice should be sought; for example, regarding renal or liver impairment, heart disease, and family history of thrombosis or cancer. GPs should be aware that suddenly stopping oestrogens may result in withdrawal, leading to menopausal-type symptoms; this may worsen any distress experienced.^2^

#### Patient's hopes for the future

It is important to ask the patient how they see the future, and whether this involves undergoing cross-sex hormonal therapy or surgery; not all patients will want these. The procedures can be discussed in more detail at a later stage if necessary.

Surgeons prefer the patient to stop smoking before, and for several months after, any procedures so it is worth discussing smoking cessation early. Other modifiable risk factors for surgical fitness (for example, obesity or hypertension) should be also addressed.^2^

### Actions advised

**Figure 1.**
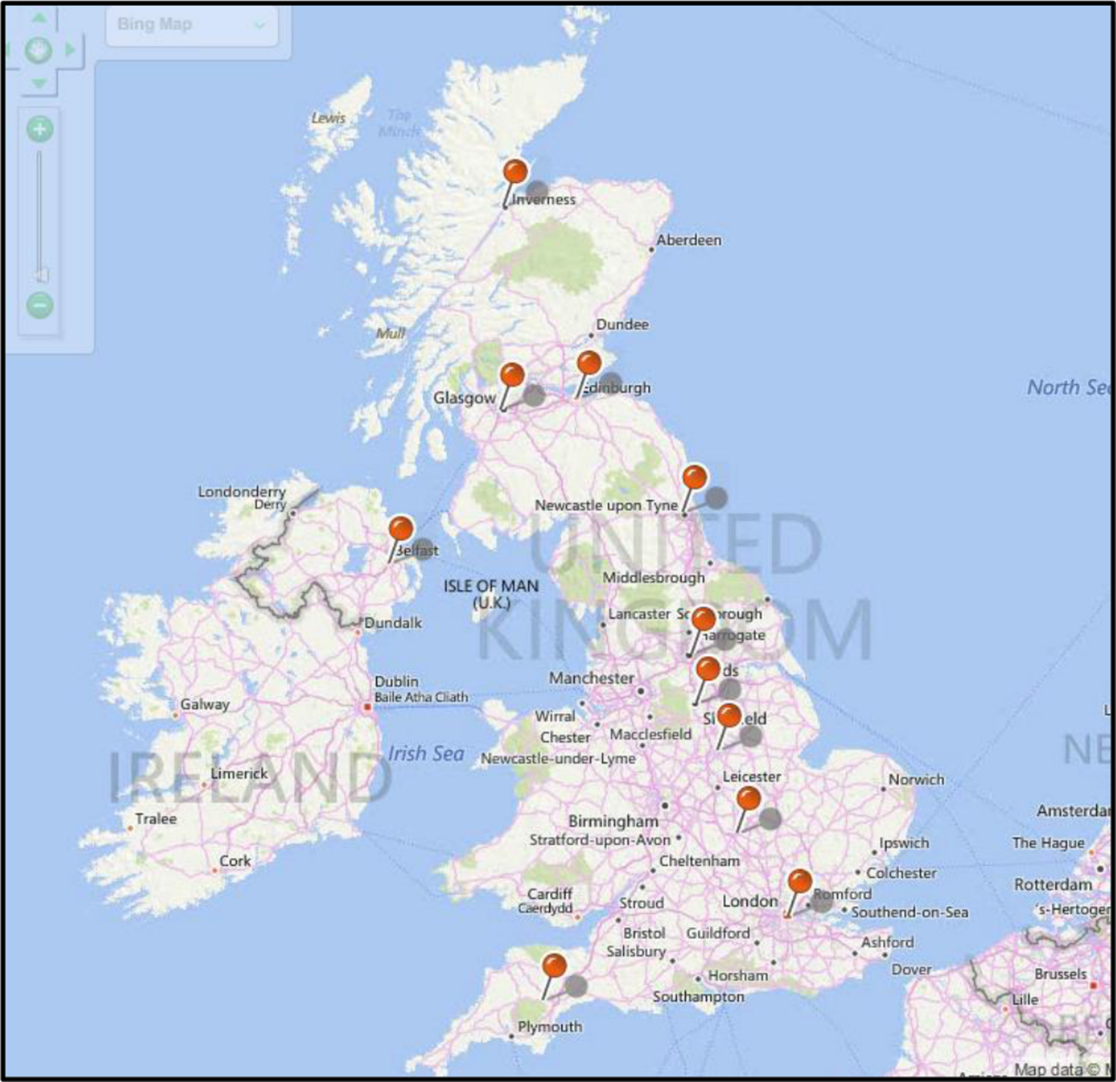
UK locations of specialist gender identity clinics.

- Ensure the patient’s electronic record is updated with the correct pronoun and patient’s desired name.
- Outline potential treatment options to include psychological therapy, speech and language therapy, hormones, and surgery.
- Discuss with the patient a direct referral to a specialist gender identity clinic (the locations of UK clinics are given in Figure 1), advising that wait times are often lengthy.
- Explain that initiating medications is usually done by the specialist gender identity team or under their advice, then discuss medication side-effects and risks (Boxes 1 and 2).
- If the individual is distressed or experiencing mental ill health, discuss a referral to the community mental health team.
- If the patient is self-medicating, consider specialist advice from an endocrinologist.
- Discuss smoking cessation if the patient is a smoker, or weight loss if they are overweight.
- Provide the individual with advice on websites or support groups for transgender people.^2,5^

**Box 1.** Side-effects of hormonal preparations for trans men and women.^2^

Trans women	Trans men
• Breast development takes 2 years	• Beard and body hair growth
• Decreased hair loss	• Male pattern baldness
• Reduced muscle bulk	• Enlarged clitoris
• Erection/orgasm harder to achieve	• Heightened libido
• Weight gain	• Acne
• Reproductive implications, such as infertility	• Sleep apnoea
	• Weight gain
	• Reproductive implications, such as infertility

**Box 2.** Risks of hormonal preparations for trans men and women.^2^

**Medications for trans women**	**Medications for trans men**	**Self-medication**
Thrombosis	Polycythaemia	Non-genuine or inactive product
Gallstones	Hyperlipidaemia	Contaminated/harmful preparation
Elevated liver enzymes	Cardiovascular disease	May have contraindications
Hypertriglyceridemia	Hypertension	Inadequate monitoring, such as liver fuction tests
Hyperprolactinaemia	Type 2 diabetes	Over- or underdosing
Type 2 diabetes		

## GMC guidance on prescribing cross-sex hormones

GMC guidance advises that there are three scenarios when it may be appropriate for GPs to ‘bridge a prescription’^6^ of cross-sex hormonal therapy until patients are seen by specialists:

- The individual is self-medicating with unverified hormonal preparations.
- Without medications, the patient experiences severe distress that may cause them to self-harm or attempt suicide. In both scenarios the GMC argues that the patient is likely to come to more harm without, rather than with, the medications. However, specialist advice is still recommended before starting hormones, leaving some ambiguity about their guidance.^7^ 
- Medications are recommended and under guidance of a specialist service, and the lowest possible dose is prescribed.

The British Medical Association’s General Practitioners Committee has expressed concern that initiating and prescribing these medications is outside of GPs’ expertise and that they have not had adequate training. Gender identity clinics have a multidisciplinary team of specialist psychologists, endocrinologists, psychiatrists, and surgeons — a combination of expertise that will not be found in general practice — so, if GPs are unsure about the best course of action, it is advisable to seek specialist advice about the lowest possible dose, before initiating hormonal therapy.^5–8^

## Follow-up

Long-term follow-up requires ongoing monitoring of hormone medications. Measuring blood pressure and blood tests at least every 6 months for the first 3 years is recommended by a number of specialist sources.^9,10^ Tests cover:

- full blood count;
- electrolytes;
- liver function;
- HbA1C
- lipids;
- testosterone;
- oestrogen;
- prolactin; and
- thyroid function.

Regular monitoring prevents any potential for peaks and troughs of hormone concentrations.^11^

Care must be taken to retain patients in appropriate national screening programmes following transition. For example, depending on surgical status, a trans man may still need regular cervical smears or mammograms but may not automatically be included for recall via electronic patient records. Be aware that trans women retain a risk of prostate cancer, and trans women who take oestrogens have a higher risk of breast cancer compared with non-transgender men. Finally, risk stratification (for instance with tools such as Q-Risk 2, FRAX) should assume gender assigned at birth and include the effects of hormonal treatments when calculating risk.^2,10^
